# Expanding the footprint of the Storegga tsunami through new evidence from Arctic marine sediments

**DOI:** 10.1038/s41598-025-10811-7

**Published:** 2025-07-10

**Authors:** Dhanushka Devendra, Magdalena Łącka, Natalia Szymańska, Hasitha Nethupul, Joanna Pawłowska, Małgorzata Szymczak-Żyła, Magdalena Krajewska, Prasadi De Silva, Stein Bondevik, Steven J. Gibbons, Marek Zajączkowski

**Affiliations:** 1 Department of Paleoceanography, Institute of Oceanology, Polish Academy of Sciences, Sopot, 81-712 Poland; 2 Department of Civil Engineering and Environmental Sciences, Western Norway University of Applied Sciences, P.O. Box 133, Sogndal, 6851 Norway; 3 Norwegian Geotechnical Institute (NGI),, P.O. Box. 3930, Ullevål Stadion, Oslo, 0806 Norway

**Keywords:** Environmental sciences, Natural hazards

## Abstract

The Storegga tsunami, dated to 8150 years ago, disturbed sea floor sediments near its source. Here, we present evidence that sediments as far north as the Kveitehola Trough (75° N) in the NW Barents Sea were affected by the Storegga tsunami. We identified an 18-cm-thick sandy mud unit, characterized by coarser grains at its lower boundary, to be deposited between 8100 and 8600 years BP. The unit contained significantly older foraminiferal tests, radiocarbon dated to between 12,000 and 27,000 years BP, that have been interpreted to be redeposited. In addition, the unit shows elevated signals of terrestrial plant sequences (sedaDNA) and terrestrial steroids. Simulation of the Storegga tsunami reveals strong currents in the shallower parts of the Barents Sea that were capable of moving sediments on the sea floor. We suggest that the tsunami backwash from the inundation of northernmost Norway or Bjørnøya could have carried terrestrial signals to the sediment core location.

**Supplementary Information:**

The online version contains supplementary material available at 10.1038/s41598-025-10811-7.

## Introduction

Large tsunamis generate currents that can disturb the seabed and rework offshore sediments. The Storegga slide, a massive submarine slope failure off the continental shelf of central Norway, triggered an extensive tsunami^1,2^ dated to 8140 ± 55 years BP^1,3^. Deposits from this giant tsunami have previously been extensively documented from Norway^4,5^, Shetland^6^, the Faroe Islands^7^, the east coast of Greenland^8^ and the north and east coasts of Scotland^9–11^. Recently, it was shown that the Storegga tsunami also disturbed and reworked offshore sediments in the North Sea and Norwegian Sea^2^.

**Fig. 1:**
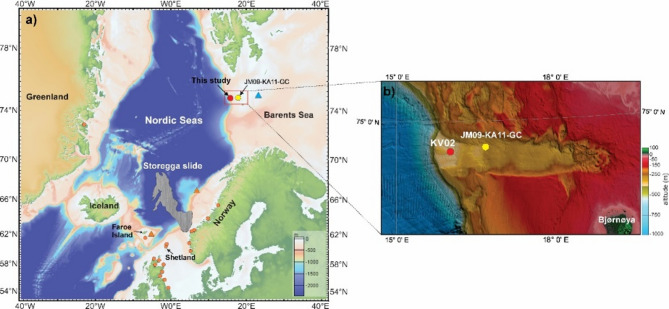
The Storegga Slide, onshore tsunami deposits, marine sediment cores at 74°N, and bathymetry of the Kveitehola Trough. (a) The red-filled circle shows the location of our sediment core (KV02). Nearest other sediment core in Kveitehola is JM09-KA11-GC^14,15^, located 18 km farther east (yellow-filled circle). The blue-filled triangle shows the additional location of the simulated time series. The Storegga landslide area^16^ is shown as a grey-filled polygon. Orange-filled circles represent onshore post-tsunami deposit locations (references are mentioned in the text), and orange-filled triangles mark offshore sediment cores that reported Storegga tsunami impacts^2^. (b) Map of Kveitehola Trough with location of sediment cores. Bathymetric maps were generated using *GeoMapApp* software version 3.7.5 (http://www.geomapapp.org)^17^.

The Storegga tsunami event coincided in time with the 8200-year BP cold event^12^ which is generally attributed to a temporary slowdown or shutdown of Atlantic Meridional Overturning Circulation (AMOC), likely triggered by the drainage of glacial Lake Agassiz^13^. Recently, Bondevik, et al.^2^ argued that the paleoclimate reconstructions based on marine sediment cores in the Nordic Seas should be reconsidered, as the cores were contaminated by Storegga tsunami deposits. Their argumentation might also apply to areas further north. Using computer simulations, Bondevik, et al.^2^ demonstrated that the Storegga tsunami reached further north than 74°N. Here, we expanded the tsunami simulations even farther north into the Barents Sea, beyond the locations of offshore sediment cores previously studied^2^ (Fig. 1a).

In this study, we simulate the wave amplitudes and velocities of the Storegga tsunami at latitudes beyond 74°N and assess possible tsunami deposits in the Kveitehola trough. Our multi-proxy analysis of a sediment core reveals that the Storegga tsunami disturbed the seabed and reworked the sediments in the northwestern Barents Sea.

### Regional setting

Kveitehola Trough is located at the western Barents Sea margin, northwest of Bjørnøya (Fig. 1). This trough is approximately 100 km long and 15–20 km wide, characterized by water depths ranging from 200 to 400 m and is surrounded by Spitsbergenbanken to the north, south, and east^14,18^ (Fig. 1b). The U-shaped cross-section of the trough, along with the presence of mega-scale glacial lineations^14,19^ indicates that Kveitehola was part of the Storfjorden glacial system, with ice streams that extended from southern Svalbard in the north and Bjørnøya in the south during the last glaciation^20,21^ and the area was completeley deglaciated by around 14 kyr BP^22^. The unlithified sediments overlying the pre-glacial bedrock in the Kveitehola region are some of the thickest found across the entire Barents Sea shelf, with local thickness of pre-Holocene deposits reaching up to 180 m^23^.

It is well established that the deeper banks in the southern Barents Sea experienced strong winnowing during the early Holocene^14^. Consequently, the absence of early Holocene sediments in the western Barents Sea is considered a regional characteristic. However, the Kveitehola Trough acted as a trap for deglacial sediments^14^ and due to its sheltered location, it potentially preserves a more complete early Holocene environmental record than other parts of the southern Barents Sea. This makes it a promising location for studying Holocene regional environmental changes and searching for possible Storegga tsunami deposits.

## Results and discussion

### Lithology and chronology

**Fig. 2:**
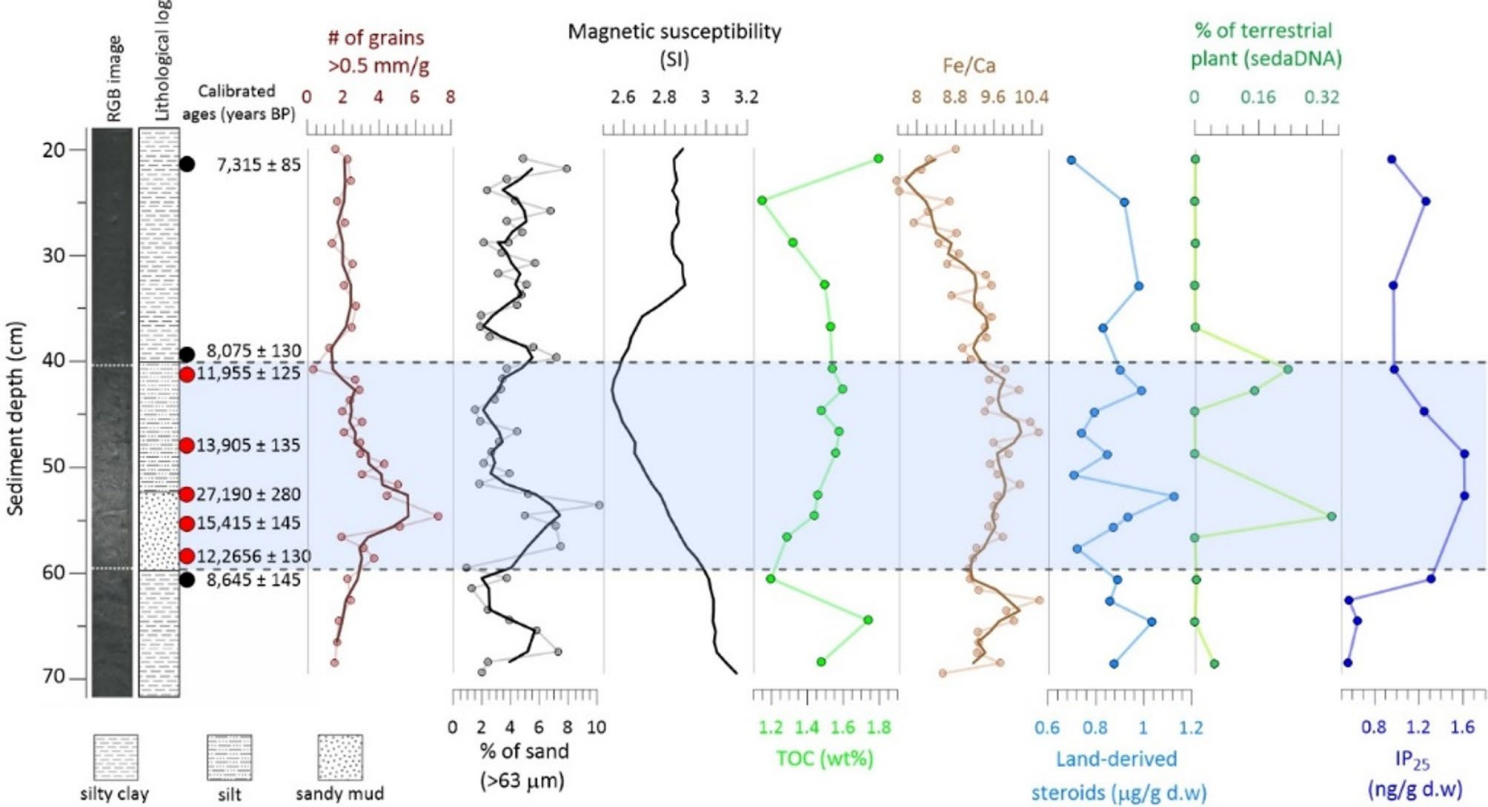
Lithology and proxies from sediment core KV02. From left to right: Line camera imagery, lithology, calibrated ^14^C dates, number of grains > 0.5 mm per gram sediments, percentage of coarse fraction > 63 μm, magnetic susceptibility, total organic carbon, X-ray fluorescence (Fe/Ca) data, land-derived steroids (sum of campesterol, β-sitosterol, stigmasterol, and sitostanol), sedaDNA relative abundances of terrestrial plants and IP_25_ concentrations. Light blue shading indicates the reworked sediment unit. In graphs where a thicker line appears, it denotes the three-point running average.

Gravity core OCE22-KV02-GC (hereafter KV02) was retrieved from the western Barents Sea (74°50.296 N, 16°1.3403E, 374 m water depth) during the AREX expedition with R/V Oceania in summer 2022 (Fig. 1). The 1.4 m-long sediment core consists of grayish, fine-grained mud (silty clay) and covers part of the deglaciation and the early and middle Holocene. This study presents results for the core interval from 20 to 70 cm depth. The visual core description, physical properties, and grain size reveal a lithologically different sediment interval between 41 and 59 cm of core depth (Fig. 2), characterized by a higher silt content compared to the silty clay sediments above and below the unit. This relatively lighter color sediment unit exhibits a coarser lower part. The high concentrations of grains > 0.5 mm and coarse-grain sediment (> 63 μm) show a fining upward grading trend within this interval (Fig. 2), reflecting an initial deposition of coarser materials followed by suspension settling that caused the upward fining.

**Table 1:** Radiocarbon measurements and calibrated ages.

Lab ID	Depth (cm)	Dated material	^14^C age	Calibrated age (year BP, 2σ)*		
				Min age	Max age	Mean age
Core KV02 (this study)						
Above redeposited unit						
11348.1.1	21	Mixed benthic foraminifera	6910 ± 80	7220	7420	7315 ± 85
11619.1.1	39	Mixed benthic foraminifera	7700 ± 125	7925	8220	8075 ± 130
Within redeposited unit						
14834.1.1	41	Mixed benthic foraminifera	10,650 ± 120^a^	11,520	12,420	11,955 ± 125
14609.1.1	48	Mixed benthic foraminifera	12,400 ± 130^a^	13,505	14,330	13,905 ± 135
11349.1.1	53	Mixed benthic foraminifera	23,720 ± 280^a^	26,560	27,705	27,190 ± 280
14610.1.1	55	Mixed benthic foraminifera	13,395 ± 140^a^	14,995	15,845	15,415 ± 145
14836.1.1	59	Mixed benthic foraminifera	10,870 ± 125^a^	11,835	12,615	12,265 ± 130
Below redeposited unit						
11620.1.1	61	Mixed benthic foraminifera	8200 ± 140	8435	8840	8645 ± 145
11350.1.1	81	Mixed benthic foraminifera	9575 ± 105	10,235	10,550	10,405 ± 110
Core JM09-KA11-GC						
Above sandy layer						
Beta-315,193	44.5	Benthic foraminifera	6890 ± 40^15^	7140	7455	7295 ± 50
Tra-1067	55	Benthic foraminifera	7630 ± 45^14^	7835	8175	8005 ± 55
Within sandy layer						
Beta-315,194	80.5	Benthic foraminifera	9140 ± 40^15^	9585	10,080	9815 ± 50
Below sandy layer						
Tra-1068	82.5	Mollusc shell	8140 ± 50^14^	8355	8775	8545 ± 60
Tra-1069	82.5	Mollusc shell	8315 ± 50^14^	8565	8995	8785 ± 60

*Calibrated AMS radiocarbon ages to calendar years of core KV02 and JM09-KA11-GC^14,15^ using the Marine20 dataset applying a regional radiocarbon reservoir offset (ΔR) of -92 ± 34 years (see “Methods”). ^a^ Reversal ages caused by tsunami currents induced sediment reworking (see below).

All five radiocarbon dates from this interval are exclusively older than those from above or below this particular unit (Table 1; Fig. 2). The inverted ages are up to about 20,000 years older than expected and clearly show the ^14^C-dated foraminifera to be reworked and redeposited (Table 1; Fig. 2). We think this sediment unit, with a coarser-grained lower layer (~ 6 cm thick), fining upward, and characterized by anomalously old ages, is a candidate to be deposited by currents in the Storegga tsunami.

### Simulations of the Storegga tsunami

**Fig. 3:**
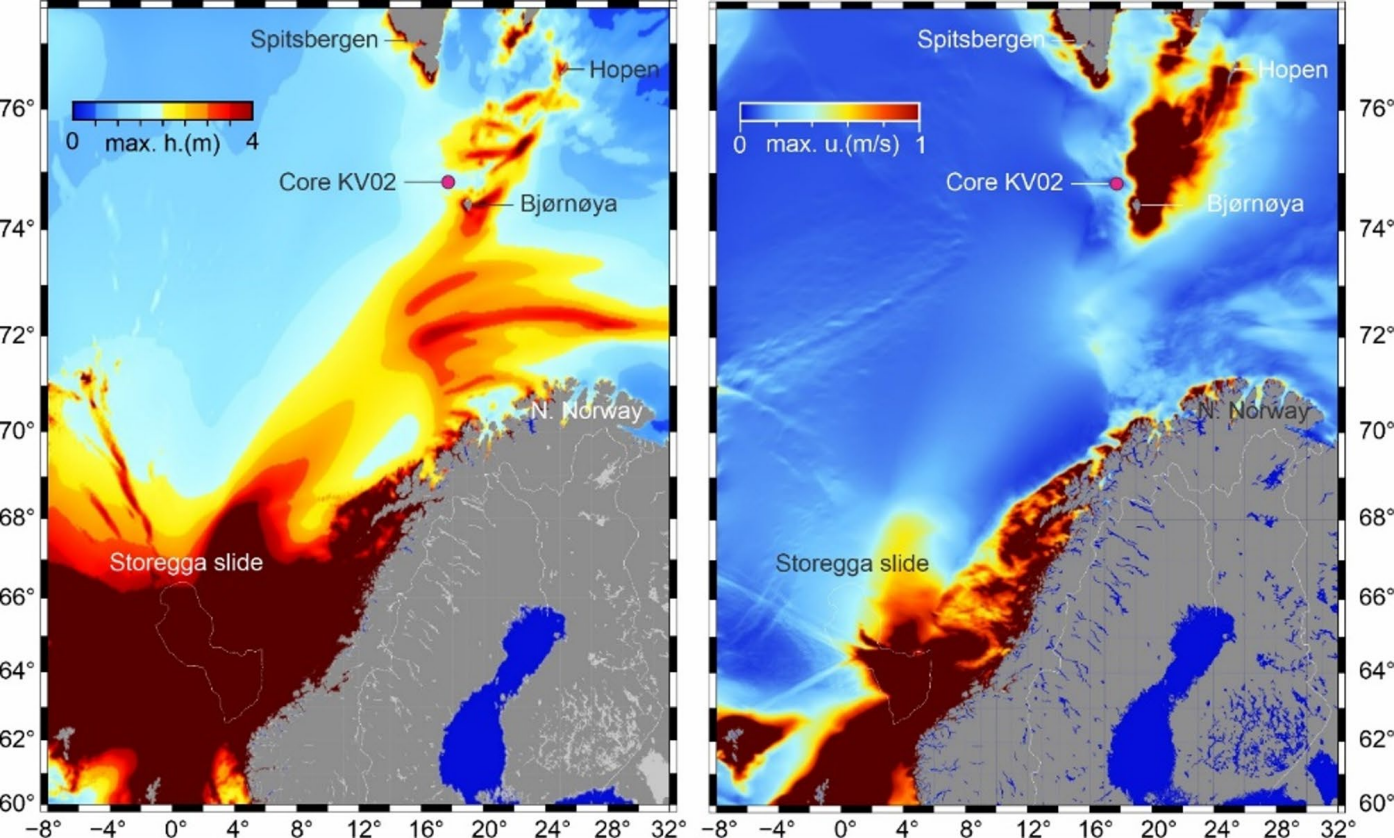
(a) Simulation of the Storegga tsunami in the European Arctic showing maximum surface elevation. Pixels in dark red-brown have maximum surface elevation > 4 m. (b) maximum flow velocity of the water column during the Storegga tsunami. Values exceeding 1 m/s are displayed with a uniform dark red/brown colour. Each pixel indicates the maximum flow velocity obtained during the 10 h simulation time. The maximum surface elevation is associated with the leading wave, which arrives at the Kveitehola Trough approximately 3 h after the onset of the slide.

We extended the Storegga tsunami simulations beyond the Kveitehola Trough and found currents > 1 m/s on the shallow Spitsbergen banks (Fig. 3). A new tsunami simulation was necessary since the domain covered by the model in Bondevik, et al.^2^ did not cover the sites of interest in this study. The new simulation was performed on a grid with 2 km lateral resolution extending from − 10° to 34° degrees East and 58° to 78° degrees North. The simulation is done with the present sea level, although the sea level was lower around Kveitehola at the time of the tsunami – for instance, at Bjørnøya, there are no elevated shorelines as are found on Spitsbergen or along the Norwegian coast^24^. However, lower sea levels would give even stronger currents than the > 1 m/s we simulated here.

For such long tsunami waves, the currents are nearly uniform from the surface to the seabed. We used the same computer model as described in Bondevik, et al.^2^ (see “Methods”). They found the current velocity 1 m above the sea floor to be reduced by 5 to 30% compared with the uniform simulated velocity in the water column – we expect similar reductions here at the seafloor compared with our simulations.

The model shows maximum wave heights of 4–5 m at the northernmost coast of Norway and near Bjørnøya (Fig. 3a). The simulations are in agreement with observations of run-up sediments in northern Norway^25^. The simulations indicate that the Storegga tsunami also reached Svalbard (Fig. 3). However, ^14^C-dated driftwood and whalebones used to reconstruct sea level changes on Svalbard do not show that they have been thrown higher up than expected around this time^26,27^, although a small run-up of 1–3 m could probably not be detected in those sea level curves.

**Fig. 4:**
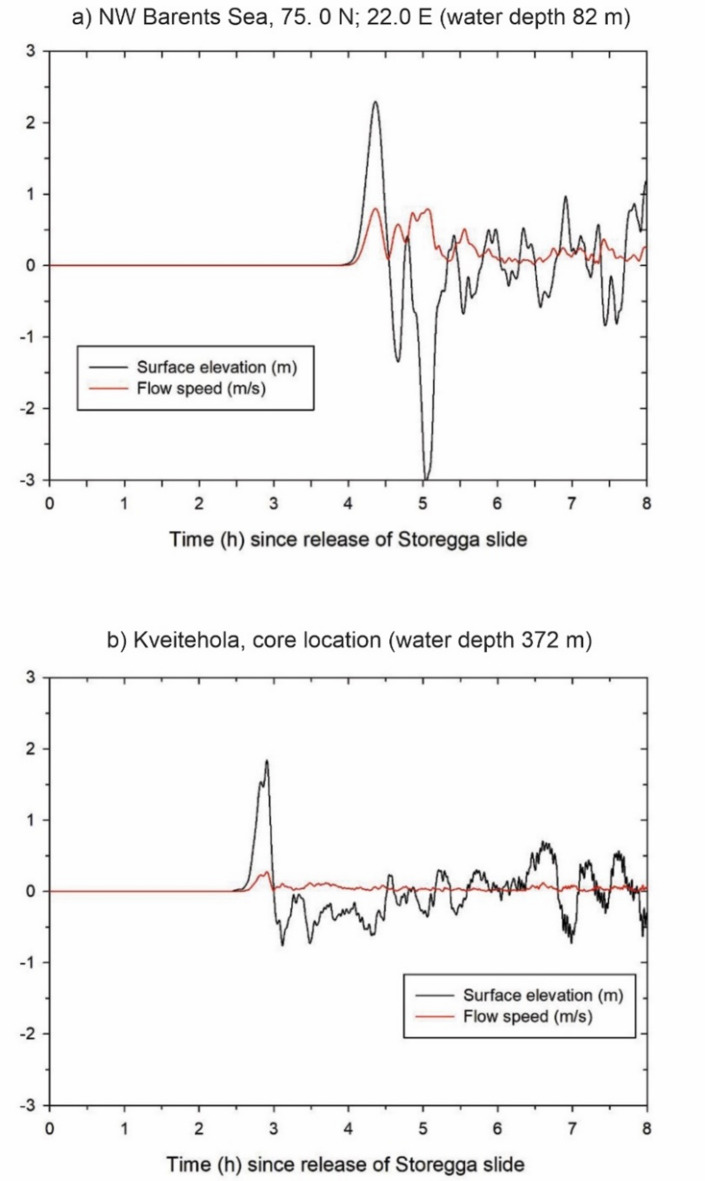
Simulation of sea surface elevation and flow velocity. (a) at the shallow bank east of Kveitehola (see blue triangle in Fig. 1), and (b) at the core location KV02 in Kveitehola. The y-axis is the same for both curves. Present water depths in brackets. The flow velocity is slightly lower at the seabed, probably reduced by a factor of somewhere between 0.7 and 0.95 of the simulated flow velocity, as shown here^2^.

Our simulation reveals that the shallower banks east of Kveitehola experienced strong Storegga currents (Fig. 3b). The water depth in this area ranges from approximately 30–100 m. At these low depths, shallower than 85 m, we calculated currents stronger than 1 m/s (Fig. 4a). Down in the Kveitehola Trough, at a depth of 370 m, only a maximum current of 0.2 m/s is modelled in the water column (Fig. 4b), probably insufficient to erode much sediment, in agreement with the radiocarbon age of 8.6 kyr BP below the layer (Fig. 2). We believe the currents eroded sand and mud on the shallower banks and transported these into the deeper parts of Kveitehola. Additionally, backwash from the northern Norwegian coast or the inundation of Bjørnøya could have introduced terrestrial materials to our study area, a hypothesis further discussed below.

### Evidence of the Storegga tsunami currents in the Northwestern Barents sea

The studied sediment core revealed a redeposited and mixed sediment interval between core depths 41–59 cm (Fig. 2). Proxies from this interval show well-defined anomalies, including changes in terrestrial signals, grain size, magnetic susceptibility, stable isotopes, and inverted radiocarbon dates.

Firstly, the redeposited unit exhibits elevated terrestrial signals, possibly carried with the tsunami backwash. The sedaDNA relative abundances of terrestrial plants (see “Methods”) show higher levels within the redeposited unit (Fig. 2). The sedaDNA signals are absent or nearly negligible throughout the rest of the analyzed sediment core (Fig. 2). The relatively low percentages of terrestrial plant sequences could likely result from using general eukaryotic primers (V9; see “Methods”). Using markers specific to terrestrial plants could provide a more comprehensive overview of the land plant community. Furthermore, this unit exhibits increased levels of land-derived steroids (terrigenous biomarkers; Fig. 2), including campesterol, β-sitosterol, stigmasterol, and sitostanol, all produced by vascular land plants^28,29^. While land-derived sterol signals remain relatively stable throughout the analyzed part of the sediment core, they show distinct changes within the redeposited unit, which correlates with sedaDNA signals. These terrestrial-originated signals are further supported by changes in Fe/Ca ratios and total organic carbon (TOC) data (Fig. 2). Although the increases are moderate, both Fe/Ca and TOC show an increasing trend from the base of the redeposited unit, following a notable decline immediately before its onset. Since Fe/Ca ratios are commonly used as indicators of terrestrial input^30,31^ this suggests an episode of enhanced land-derived sediment transport.

Our tsunami simulations indicate a wave amplitude of at least 4 m around the northern Norwegian coast and Bjørnøya (Fig. 3), suggesting that tsunami waves likely inundated these coastal areas. We propose that Storegga tsunami-induced return currents (backwash) transported these terrestrial materials from the run-up areas on the northern Norwegian coast or the northwestern coast of Bjørnøya. Alternatively, these terrestrial materials within the redeposited unit may result from the resuspension and redeposition of older sediments, potentially introduced to the site from the shallower bank.

Secondly, the lower boundary of the redeposited unit is composed of coarser grains and upper 11–12 cm of the unit shows a fining upward grading trend (Fig. 2). Similar coarser sediment layers have been documented in offshore sediment deposits following the Storegga tsunami in the Norwegian Sea^2^ and are common in cores from coastal lakes inundated by Storegga tsunami waves^32^. Sorted sandy layers and a fining upward trend akin to our observation were identified in deposits after the 2011 Tohoku tsunami in Japan^33^.

Thirdly, the foraminiferal species identified in this reworked sediment unit are poorly preserved and occur in low counts. A higher abundance of *Elphidium clavatum* and larger test-bearing *Cibicidoides lobatulus* correlate with coarser sediment and lower magnetic susceptibility (Supplementary Fig. 1). Knudsen, et al.^34^ documented a similar distinct minimum in magnetic susceptibility, accompanied by a peak of chronologically old *C. lobatulus* tests between 8200 and 8000 years BP from the North Icelandic shelf. They also attributed this pattern to sediment reworking. Bondevik, et al.^2^ considered this evidence and suggested that the Storegga tsunami likely caused the reworking.

Finally, the gradual but notable decline in magnetic susceptibility through this part of the record suggests a sudden change in sediment transport to the area (Fig. 2). This shift is accompanied by the distinct increase in IP_25_ concentrations, an established indicator of sea ice presence, compared to the sediments above and below the redeposited unit (Fig. 2), which is mostly composed of late glacial and deglacial sediments, as suggested by radiocarbon ages (Table 1). This suggests the deposition of older sediments containing a sea-ice proxy signal. In addition to the sea ice proxy signal, this redeposited unit also exhibits changes in oxygen isotope values (δ^18^O) of benthic foraminiferal species, *E. clavatum*, which decrease from 3.51‰ (61 cm) to 3.04‰ (55 cm) and 3.05‰ (53 cm) within the coarser sediment unit (Supplementary Fig. 1). These lower δ^18^O values suggest that low salinity bottom waters prevailed when the foraminiferal tests were being calcified. Radiocarbon dating of these lighter δ^18^O foraminifera yielded ages of 15,415 ± 145 and 27,190 ± 280 years BP, respectively (Table 1; Supplementary Fig. 1). The lighter δ^18^O and presence of IP_25_ likely reflect meltwater influence near the ice margin, where both sea ice presence and freshwater input could coexist. This interpretation supports the idea that the redeposited sediments were originally deposited in an ice-proximal setting.

The distance from our study site to the Storegga slide is about 1250 km, which has been shown to be a realistic propagation range^35^. The computer simulation suggests that the tsunami generated strong flows as far north as 74°N^2^ with the potential to disrupt offshore sediment (Fig. 3). While we do not observe a distinct erosional base in our redeposited sediment unit aside from a coarser sandy layer, our interpretation is supported by findings of Rüther, et al.^14^ who identified a similar sandy layer with a well-developed erosional base, dated to approximately 8200 years BP in core JM09-KA11-GC, collected ~ 30 km east of our study site at a water depth of 345 m (Fig. 1). They suggested that the erosion was most likely caused by Storegga tsunami-generated waves. On the other hand, an old radiocarbon date from this sandy layer of the same core JM09-KA11-GC was used in climate reconstruction by Berben, et al.^15^ which contrasts with radiocarbon dates from the adjacent sediments (Table 1). Berben, et al.^15^ incorporate this older radiocarbon date into their age model while discarding the younger date below the sandy layer (Table 1). We think this approach could be problematic, as the older radiocarbon age reflects the redeposition of older material rather than an in situ depositional event. Instead, the younger age should be used as it represents the pre-tsunami sedimentation process and avoids the influence of reworked older material.

## Conclusions

The sedimentary record from core KV02 reveals a distinct unit of reworked sediment with anomalous characteristics, providing strong evidence for a significant disturbance event between 8100 and 8600 years ago, which we attribute to the Storegga tsunami (dated to 8150 +/40). This conclusion is supported by a suite of proxy indicators, including a notable decrease in magnetic susceptibility, a coarser lower boundary, and a fining upward grading trend within the reworked unit. Our tsunami simulations, along with elevated signals of terrestrial plant sequences (sedaDNA) and terrestrial steroids, suggest that the tsunami reached and washed over the Norwegian coast and/or Bjørnøya, transporting terrestrial material to the northwestern Barents Sea. These findings provide factual evidence that the Storegga tsunami impacts extended farther north than previously recognized.

## Methods

### Core handling

Sediment core OCE22-KV02-GC was opened under sterile conditions in the sedimentary ancient DNA-dedicated laboratory at the Institute of Oceanology, Polish Academy of Sciences (IOPAN). After the sediment core opening, the visual description was performed, and color information was obtained based on the Munsell Soil Color Chart. The sediment color varies mostly between olive-gray and grayish-green, with compositions ranging from silty clay to sandy mud. The selected core sections (20–70 cm) from the 1.42 m long core was sliced at 1 cm intervals, freeze-dried, and wet sieved using sieves with a 63, 100, and 500 μm mesh size.

### AMS-^14^C dating

The nine radiocarbon dates were obtained from specimens of the mixed benthic foraminifera sample dated at the MICADAS facility at the Alfred-Wegener Institute in Bremerhaven, Germany (Fig. 2; Table 1). The radiocarbon ages from OCE22-KV02-GC and the presented radiocarbon dates from JM09-KA11-GC (Table 1) were calibrated to calendar ages in the CALIB ^14^C software (8.2.0; Stuiver, et al.^36^) using the Marine20 dataset^37^ and applying a regional radiocarbon reservoir offset (ΔR) of -92 ± 34 years. This ΔR value of − 92 ± 34 years corresponds to 67 ± 34 years relative to the Marine04 calibration curve based on data from near Bjørnøya^38^. We used http://calib.org/marine/ (last accessed 2024/04/08) to obtain the ΔR value relative to the Marine20 calibration curve.

### Foraminiferal analyses

Foraminiferal analyses were performed on specimens from the > 100 μm fraction. The abundance of planktic foraminifera was very low throughout the analyzed core section. When necessary, residues were split using a dry microsplitter, and the total number of benthic foraminifera was calculated. Picked foraminifera were identified under a stereo-microscope. The taxonomic classification was performed mostly at the species level, using the generic classification of Loeblich Jr and Tappan^39^.

### Stable isotopes

Oxygen and carbon stable isotope compositions of tests of the infaunal foraminifer species *Elphidium clavatum* were determined. All samples were cleaned in methanol, and measurements were performed using a Thermo Finnigan MAT 252 mass spectrometer with a Kiel III automatic carbonate preparation device at the Light Stable Isotope Mass Spec Laboratory, Department of Geological Sciences, University of Florida, USA. Measurement precision was better than ± 0.04‰ and ± 0.02‰ for oxygen and carbon isotopes, respectively.

### Biomarker analysis

Biomarkers (IP_25_ and polar steroids) were extracted from sediments and analyzed using GC/MS according to the procedure described in detail elsewhere^40,41^. Briefly, the freeze-dried sediment sample spiked with the surrogate standards (9-octylheptadecene, 7-hexylnonadecane, and androstanol) was sonication-extracted with a dichloromethane: methanol (2:1 v/v) mixture. After extraction, the raw extract was concentrated by rotary evaporation and divided into two subsamples (A and B). Subsample A was fractionated by solid-phase extraction (SPE) using 1% deactivated silica gel. The fraction containing IP_25_ was eluted with a hexane and dichloromethane (1:1 v/v) mixture. To determine polar steroids, subsample B was saponified with 5% KOH in methanol, and then liquid-liquid extraction with chloroform was performed. The combined chloroform fractions were concentrated by rotary evaporation and derivatized to produce the trimethylsilyl derivatives. Biomarkers were analyzed using a gas chromatograph coupled to a mass spectrometer detector (GCMS-QP2010 Ultra; Shimadzu) according to the procedure described in Szymczak-Żyła and Lubecki^40^ and Krajewska, et al.^41^.

### Grain size analysis

Sediment grain size analyses (0.02–2000 μm) were performed using a Malvern Mastersizer 2000 Particle Size Analyzer at the IOPAN. Before analysis, each sediment samples were pretreated with excess hydrogen peroxide (H_2_O_2_; 30%) and HCl (10%) for 24 h to remove organic matter and carbonates, respectively. The resulting organic matter and carbonate-free samples were diluted using distilled water and fully dispersed before measurement. The grain size data obtained were processed using the GRADISTAT™ software version 8.0^42^.

### Organic bulk sediment parameters

Total organic carbon (TOC) was measured on homogenized bulk sediment samples using the combustion technique with chromatographic detection, performed with a Thermo Flash 2000 elemental analyzer. The TOC measurements were performed after removing residual carbonate by adding hydrochloric acid.

### X-ray fluorescence spectrometry

We measured the relative elemental composition of the core segment using an Olympus Vanta M series portable X-ray fluorescence (XRF) analyzer at the Department of Paleoceanography, IOPAN. The core surface was covered with 4 μm polypropylene film to reduce contaminations. The instrument uses a Rh anode X-ray tube (8–50 kV) as the excitation source. The instrument was operated in Geochem Mode with a scanning time of 45 s per beam. The instrument completed one whole scan in 90 s by scanning via two beams in sequence. For the interpretation, element ratios, rather than individual elements, are used to prevent closed-sum effects.

### Sedimentary ancient DNA (sedaDNA)

Samples for sedimentary ancient DNA (sedaDNA) analyses were taken at 2 cm intervals, and approximately 10 g of sediment was obtained from each layer. The sediment was transferred to sterile containers and stored at – 20 °C until further analysis.

Total DNA was extracted from 10 g of sediment using the DNeasy PowerMax Soil Kit (Qiagen) according to the manufacturer’s instructions. The V9 region of the eukaryotic 18S SSU rDNA gene was amplified by PCR using V9_1389F-B forward and V9_1510R reverse primers^43^. The primers were labeled with a unique combination of 8 nucleotides attached to their 5’ ends to allow further demultiplexing. Three PCR replicates were prepared for each sample and each reaction was run in a total volume of 25 µL containing 1.5 µL 1.5 mM MgCl_2_ (Applied Biosystems, USA), 2.5 µL 10× PCR Buffer II (Applied Biosystems), 0. 5 µL of 0.2 mM deoxynucleotide triphosphates (Promega, USA), 0.5 µL of 20 mg/mL bovine serum albumin (Invitrogen Ultrapure, USA), 1 µL of 10 µM of each primer, 0.2 µL of AmpliTaq Gold DNA polymerase (Applied Biosystems) and 2 µL of template DNA. Amplification conditions consisted of pre-denaturation at 95 °C for 5 min, followed by 50 cycles of denaturation at 95 °C for 30 s, annealing at 57 °C for 1 min, and extension at 72 °C for 1:30 min, followed by a final extension step at 72 °C for 5 min. PCR products were verified by agarose gel electrophoresis, purified using the Clean-Up kit (A&A Biotechnology, Poland), and quantified using the QuantusTM fluorometer (Promega, USA). Amplicons were pooled in equimolar concentration within each multiplexed library. Libraries were quantified by quantitative PCR using the Kapa Library Quantification kit (Kapa Biosciences, USA) and sequenced on a NovaSeq 6000 instrument (Illumina, USA) in paired-end reading mode 2 × 150 with Kit v1.5 (300 cycles) at the Genomics Core Facility, Center of New Technologies, University of Warsaw.

Raw sequence reads were analyzed using the web application SLIM^44^. First, sequencing reads were demultiplexed based on the unique tag combinations using the module demultiplexer. Quality filtering, chimera removal, and amplicon sequence variants (ASVs) generation were performed using DADA2 v.1.16^45^ with a pseudo pool parameter. Furthermore, ASVs occurring in only one sample and rare ASVs (with less than 10 reads) were removed. The remaining ASVs were curated using the LULU package v.0.1.0^46^ with the parameter settings of minimum match = 97 and minimum relative co-occurrence = 0.9. ASVs were assigned using VSEARCH^47^ against the PR2 database v.4.14.1^48^. The unassigned ASVs were blasted against the NCBI databases^49^. Finally, the ASVs assigned to terrestrial plants (Embryophyceae) were presented as an ASV-to-sample table, and the abundance of each ASV was expressed as a percentage of the total sequence reads [%].

### Computer simulation of the Storegga tsunami

The tsunami wave propagation is modelled using a two-stage coupled landslide and tsunami model. The landslide is modelled using the BingCLAW code for cohesive landslide dynamics (e.g. Kim, et al.^50^), and the tsunami simulation, driven by the seafloor displacement from the landslide, is performed using the GloBouss model, which allows for wavelength-dependent wave speed (frequency dispersion). The procedure followed was identical to that described in Bondevik, et al.^2^ and we refer to this publication for technical details of the simulation and references. A total simulation time of 10 h was applied, although the model confirms that the leading water wave leads to the highest surface elevation at the site of interest, approximately 3 h after the slide initiation.

The simulation used contemporary bathymetry obtained from the GEBCO Compilations Group, 2024 (https://www.gebco.net/data_and_products/gridded_bathymetry_data/).

## Electronic supplementary material

Below is the link to the electronic supplementary material.

Supplementary Material 1

Supplementary Material 2

## Data Availability

The datasets generated and/or analyzed during the current study are available in the PANGAEA repository (https://www.pangaea.de/).
