# Supplementary material for: Expanding the footprint of the Storegga tsunami through new evidence from Arctic marine sediments
Source: Sci Rep. 2025 Jul 10;15:24809. doi: 10.1038/s41598-025-10811-7 (PMC12246226; doi:10.1038/s41598-025-10811-7)
Supplement: Supplementary file 1 — Supplementary Material 1 [file 41598_2025_10811_MOESM1_ESM.pdf]

Table 1 | The sedimentary ancient DNA percentages of terrestrial plant, terrestrial sterols and IP_{25} concentrations

Sediment layer (cm)	sedaDNA % of terrestrial plant	Terrestrial sterols (μg g^{-1} d.w)	IP_{25} concentration (ng g^{-1} d.w)
19	NA	0.7669	1.528657
21	0.00	0.6978	0.965136
25	0.00	0.9181	1.269942
29	0.00	N/A	N/A
33	0.00	0.9782	0.980194
37	0.00	0.8285	NA
**41***	**0.23**	**0.9002**	**0.982957**
**43***	**0.15**	**0.9893**	**N/A**
**45***	**0.00**	**0.7923**	**1.255307**
**47***	**N/A**	**0.7412**	**N/A**
**49***	**0.00**	**0.8483**	**1.617205**
**51***	**N/A**	**0.7061**	**N/A**
**53***	**N/A**	**1.1246**	**N/A**
**55***	**0.34**	**0.9316**	**1.621777**
**56***	**N/A**	**0.8731**	**N/A**
**57***	**0.00**	**N/A**	**N/A**
**58***	**N/A**	**0.7221**	**N/A**
**59***	**N/A**	**N/A**	**N/A**
61	0.00	0.8893	1.317217
63	N/A	0.8586	0.577344
65	0.00	1.0344	0.65642
69	0.05	0.8738	0.566152
71	N/A	0.8023	0.861978
73	0.00	0.8646	0.865788

*layers within the redeposited sediment layer.
