# Supplementary material for: Expanding the footprint of the Storegga tsunami through new evidence from Arctic marine sediments
Source: Sci Rep. 2025 Jul 10;15:24809. doi: 10.1038/s41598-025-10811-7 (PMC12246226; doi:10.1038/s41598-025-10811-7)
Supplement: Supplementary file 2 — Supplementary Material 2 [file 41598_2025_10811_MOESM2_ESM.pdf]

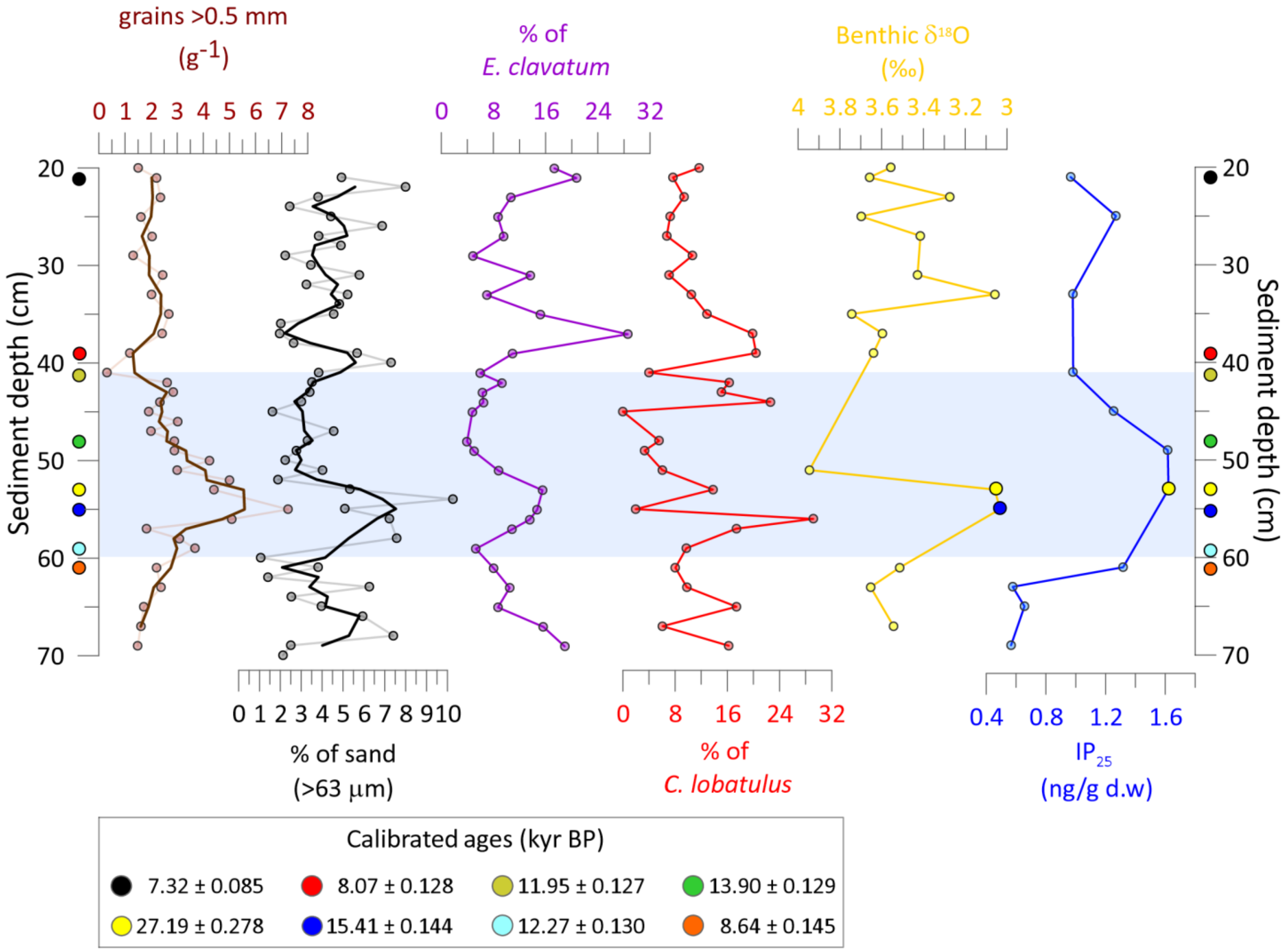

Supplementary Figure 1. | Combined records of bulk parameters, foraminiferal, stable oxygen isotope, and sea ice proxy from core KV02. From left to right: Concentrations of grains >0.5 mm, percentage of sand (>63 µm), percentage of *Elphidium clavatum*, percentage of *Cibicides lobatulus*, benthic foraminiferal $\delta^{18}O$, and IP_{25} concentrations. Light blue shading indicates a redeposited layer. Symbols next to the left and right y-axes indicate AMS ^{14}C dates.
